# Obituary

**Published:** 1919-05

**Authors:** 


### OBITUARY

DR. GEORGE CUNNINGHAM, of Cambridge, England. There has just passed away, at the age of sixty-seven, a member of the profession who was a remarkable character. A man of tremendous energy, wide interests and mental acuteness, Cunningham's work for the dental profession and for mankind was such as to compel admiration, although it was sometimes marred by defects of temperament which are perhaps inseparable from the qualities which he possessed.

His earlier professional career was one of marked success, and his location at Cambridge, where he graduated as Master of Arts, brought him in contact with many men of science and of public spirit, some of whom he infected with his enthusiasm and who did their utmost to assist him in his work of bringing the subject of dental hygiene, and especially the care of the mouths of children, before the public and the government. Notable among these was Dr. Sedley Taylor, of Trinity College, Cambridge, whose generosity made possible the experiment at Cambridge which was the pioneer of the many school clinics subsequently started. Dr. Taylor pays the following tribute to Dr. Cunningham:

"I can not allow the death of Dr. George Cunningham to pass without saying once more how essential to the success of the Cambridge Dental Clinic was the work done by him during its establishment and early progress. As professional dental adviser, he brought to bear unique knowledge, great organising power, and remarkable fertility of resource. Without the aid of these qualities the efforts of a mere lay founder, such as the writer of this letter, might easily have issued in failure."

Dr. Cunningham's first public appointment in London was as Lecturer on Operative Dental Surgery, at the National Dental Hospital. He was not an ideal lecturer, being two long and discursive, while he was always a difficult man on Committees. He could not easily see other people's point of view, and this made working with him not always a smooth matter, so it is not surprising that his tenure of the post of Dental Surgeon to the London Hospital (probably the only general hospital in London where the Dental Surgeon was not medically qualified) was a short one and that his scheme of an Institute of Dental Technology—an admirable scheme if it had been properly carried out—was doomed to failure.

Nearly thirty years ago when the increase of phosphorus necrosis among match-makers had created much agitation among the public the Government retained Dr. Cunningham to examine into the matter from the scientific dental standpoint. He visited factories in this country and abroad, and submitted exhaustive reports, which are to be found in the Blue-book containing the Report of the Select Committee of 1899. This committee recommended the gradual prohibition of yellow phosphorus in match-making, and it was undoubtedly due to the assistance given by Dr. Cunningham that the agitation was strong enough to force the Government to pass legislation which has extinguished "phossy jaw."

George Cunningham was a born enthusiast and whether it was the International Dental Federation, Esperanto, Cinematograph films to educate the public on dental matters, or as lecturer to the public, to factory hands, or to our soldiers on active service, inventing a new game of garden golf or a gas mask, he was always enthusiastic himself and the cause of enthusiasm in others. Of late years his practice had suffered and he received a pension from the Civil List for services rendered to the state, and on account of his straightened circumstances. In spite of his drawbacks—and who is perfect?—George Cunningham has earned his meed of lasting fame and will be kindly remembered by his colleagues. Above all, he has earned the blessings of the poor—the poor child and the poor match-maker—and this may comfort his soul in the Great Beyond where the scale of values does not depend on worldly success. Dr. Cunningham had many friends in the United States.

